# Prevalence and Early Identification of Autism Spectrum Disorder Among Children Aged 4 and 8 Years — Autism and Developmental Disabilities Monitoring Network, 16 Sites, United States, 2022

**DOI:** 10.15585/mmwr.ss7402a1

**Published:** 2025-04-17

**Authors:** Kelly A. Shaw, Susan Williams, Mary E. Patrick, Miguel Valencia-Prado, Maureen S. Durkin, Ellen M. Howerton, Christine M. Ladd-Acosta, Elise T. Pas, Amanda V. Bakian, Paige Bartholomew, Nancy Nieves-Muñoz, Kate Sidwell, Amy Alford, Deborah A. Bilder, Monica DiRienzo, Robert T. Fitzgerald, Sarah M. Furnier, Allison E. Hudson, Olivia M. Pokoski, Lindsay Shea, Sarah C. Tinker, Zachary Warren, Walter Zahorodny, Hilcon Agosto-Rosa, Joshua Anbar, Katheleen Y. Chavez, Amy Esler, Allison Forkner, Andrea Grzybowski, Azza Hagel Agib, Libby Hallas, Maya Lopez, Sandy Magaña, Ruby H.N. Nguyen, Jaylaan Parker, Karen Pierce, Tyra Protho, Hilda Torres, Sandra B. Vanegas, Alison Vehorn, Minyu Zhang, Jennifer Andrews, Felicia Greer, Jennifer Hall-Lande, Dedria McArthur, Madison Mitamura, Angel J. Montes, Sydney Pettygrove, Josephine Shenouda, Carolyn Skowyra, Anita Washington, Matthew J. Maenner

**Affiliations:** ^1^National Center on Birth Defects and Developmental Disabilities, CDC, Atlanta, Georgia; ^2^Puerto Rico Department of Health, San Juan, Puerto Rico; ^3^University of Wisconsin, Madison, Wisconsin; ^4^Johns Hopkins Bloomberg School of Public Health, Baltimore, Maryland; ^5^University of Utah Huntsman Mental Health Institute, Salt Lake City, Utah; ^6^University of California, San Diego, California; ^7^Rutgers New Jersey Medical School, Newark, New Jersey; ^8^Pennsylvania Department of Human Services, Harrisburg, Pennsylvania; ^9^Washington University in St. Louis School of Medicine, St. Louis, Missouri; ^10^University of Arkansas for Medical Sciences, Little Rock, Arkansas; ^11^Vanderbilt University Medical Center, Nashville, Tennessee; ^12^Arizona State University, Tempe, Arizona; ^13^City of Laredo Public Health Department, Laredo, Texas; ^14^University of Minnesota, Minneapolis, Minnesota; ^15^Indiana Department of Health, Indianapolis, Indiana; ^16^University of Texas at Austin, Austin, Texas; ^17^University of Arizona, Tucson, Arizona

## Abstract

**Problem/Condition:**

Autism spectrum disorder (ASD).

**Period Covered:**

2022.

**Description of System:**

The Autism and Developmental Disabilities Monitoring Network is an active surveillance program that estimates prevalence and characteristics of ASD and monitors timing of ASD identification among children aged 4 and 8 years. In 2022, a total of 16 sites (located in Arizona, Arkansas, California, Georgia, Indiana, Maryland, Minnesota, Missouri, New Jersey, Pennsylvania, Puerto Rico, Tennessee, Texas [two sites: Austin and Laredo], Utah, and Wisconsin) conducted surveillance for ASD among children aged 4 and 8 years and suspected ASD among children aged 4 years. Surveillance included children who lived in the surveillance area at any time during 2022. Children were classified as having ASD if they ever received 1) an ASD diagnostic statement in a comprehensive developmental evaluation, 2) autism special education eligibility, or 3) an ASD *International Classification of Diseases, Ninth Revision* (ICD-9) code in the 299 range or *International Classification of Diseases, Tenth Revision* (ICD-10) code of F84.0, F84.3, F84.5, F84.8, or F84.9. Children aged 4 years were classified as having suspected ASD if they did not meet the case definition for ASD but had an evaluator’s suspicion of ASD documented in a comprehensive developmental evaluation.

**Results:**

Among children aged 8 years in 2022, ASD prevalence was 32.2 per 1,000 children (one in 31) across the 16 sites, ranging from 9.7 in Texas (Laredo) to 53.1 in California. The overall observed prevalence estimate was similar to estimates calculated using Bayesian hierarchical and random effects models. ASD was 3.4 times as prevalent among boys (49.2) than girls (14.3). Overall, ASD prevalence was lower among non-Hispanic White (White) children (27.7) than among Asian or Pacific Islander (A/PI) (38.2), American Indian or Alaska Native (AI/AN) (37.5), non-Hispanic Black or African American (Black) (36.6), Hispanic or Latino (Hispanic) (33.0), and multiracial children (31.9). No association was observed between ASD prevalence and neighborhood median household income (MHI) at 11 sites; higher ASD prevalence was associated with lower neighborhood MHI at five sites.

Record abstraction was completed for 15 of the 16 sites for 8,613 children aged 8 years who met the ASD case definition. Of these 8,613 children, 68.4% had a documented diagnostic statement of ASD, 67.3% had a documented autism special education eligibility, and 68.9% had a documented ASD ICD-9 or ICD-10 code. All three elements of the ASD case definition were present for 34.6% of children aged 8 years with ASD.

Among 5,292 (61.4% of 8,613) children aged 8 years with ASD with information on cognitive ability, 39.6% were classified as having an intellectual disability. Intellectual disability was present among 52.8% of Black, 50.0% of AI/AN, 43.9% of A/PI, 38.8% of Hispanic, 32.7% of White, and 31.2% of multiracial children with ASD. The median age of earliest known ASD diagnosis was 47 months and ranged from 36 months in California to 69.5 months in Texas (Laredo).

Cumulative incidence of ASD diagnosis or eligibility by age 48 months was higher among children born in 2018 (aged 4 years in 2022) than children born in 2014 (aged 8 years in 2022) at 13 of the 15 sites that were able to abstract records. Overall cumulative incidence of ASD diagnosis or eligibility by age 48 months was 1.7 times as high among those born in 2018 compared with those born in 2014 and ranged from 1.4 times as high in Arizona and Georgia to 3.1 times as high in Puerto Rico. Among children aged 4 years, for every 10 children meeting the case definition of ASD, one child met the definition of suspected ASD.

Children with ASD who were born in 2018 had more evaluations and identification during ages 0–4 years than children with ASD who were born in 2014 during the 0–4 years age window, with an interruption in the pattern in early 2020 coinciding with onset of the COVID-19 pandemic.

Overall, 66.5% of children aged 8 years with ASD had a documented autism test. Use of autism tests varied widely across sites: 24.7% (New Jersey) to 93.5% (Puerto Rico) of children aged 8 years with ASD had a documented autism test in their records. The most common tests documented for children aged 8 years were the Autism Diagnostic Observation Schedule, Autism Spectrum Rating Scales, Childhood Autism Rating Scale, Gilliam Autism Rating Scale, and Social Responsiveness Scale.

**Interpretation:**

Prevalence of ASD among children aged 8 years was higher in 2022 than previous years. ASD prevalence was higher among A/PI, Black, and Hispanic children aged 8 years than White children aged 8 years, continuing a pattern first observed in 2020. A/PI, Black, and Hispanic children aged 8 years with ASD were also more likely than White or multiracial children with ASD to have a co-occurring intellectual disability. Identification by age 48 months was higher among children born in 2018 compared with children born in 2014, suggesting increased early identification consistent with historical patterns.

**Public Health Action:**

Increased identification of autism, particularly among very young children and previously underidentified groups, underscores the increased demand and ongoing need for enhanced planning to provide equitable diagnostic, treatment, and support services for all children with ASD. The substantial variability in ASD identification across sites suggests opportunities to identify and implement successful strategies and practices in communities to ensure all children with ASD reach their potential.

## Introduction

Autism spectrum disorder (ASD) is a developmental disability characterized by difficulties with social interaction or communication and the presence of restricted interests or repetitive behaviors. ASD is recognized as a heterogenous condition with wide variation in the type and severity of signs, symptoms, and levels of support needed among persons with ASD (*1*). In addition to developmental surveillance, the American Academy of Pediatrics recommends that pediatric care providers screen all children for ASD at ages 18 and 24 months (*2*,*3*). Additional screening might be needed if a child is at high risk for ASD or if signs and symptoms are present. Early identification of ASD can help children receive services and supports they might need for their development and to improve long-term outcomes (*4*).

The Autism and Developmental Disabilities Monitoring (ADDM) Network has reported biennial ASD estimates among children aged 8 years since 2000. Prevalence increased from one in 150 in 2000 (*5*) to one in 36 in 2020 (*6*), and demographic patterns in ASD identification changed. Before 2016, the highest ASD prevalence was observed among White children and in children from neighborhoods with higher socioeconomic status (SES) (*7*). In 2020, higher ASD prevalence was observed for the first time among historically underserved groups including non-Hispanic Black and Hispanic children, and an association between ASD prevalence and SES (measured by median household income [MHI] tertile) was not present in the majority of sites (*6*).

The ADDM Network began tracking ASD among children aged 4 years in 2010 as an indicator of early identification of ASD (*8*). Although prevalence has been lower among children aged 4 years than aged 8 years in each reporting year, comparing cumulative incidence of identification by age 48 months has consistently indicated higher rates of ASD identification in younger cohorts (i.e., children born more recently) (*9*–*11*). A report that presented data collected in 2020 found interruptions to early identification during the onset of the COVID-19 pandemic (*11*).

This report includes data from the expansion of the ADDM Network to 16 communities across the United States monitoring children aged 4 and 8 years with ASD in 2022. The ADDM Network began its sixth funding cycle in January 2023 with 11 sites; five additional sites were able to join in April because of 2023 Consolidated Appropriations Act (*12*) funds for expansion.

This report describes prevalence and characteristics of children with ASD as well as patterns in early ASD identification. These data can be used by communities to monitor trends, anticipate and understand service needs, and support efforts to ensure early and equitable identification of children with ASD.

## Methods

### Surveillance Sites and Procedures

ADDM Network sites in Arizona, Arkansas, California, Georgia, Indiana, Maryland, Minnesota, Missouri, New Jersey, Pennsylvania, Puerto Rico, Tennessee, Texas (two sites: Austin and Laredo), Utah, and Wisconsin selected a geographic area of their state to conduct surveillance of ASD among children aged 4 and 8 years and surveillance of suspected ASD among children aged 4 years in 2022. All sites functioned as public health authorities under the Health Insurance Portability and Accountability Act of 1996 Privacy Rule and met applicable local institutional review board, privacy, and confidentiality requirements under 45 CFR Part 46. Sites also functioned as authorized representatives of Individuals with Disabilities Education Act (IDEA) agencies to access education records under Family Education Rights and Privacy Act and IDEA consistent with 34 CFR Section 99.35.

### Case Ascertainment and Surveillance Case Definition

Surveillance was conducted using the same surveillance methods and case definitions used in 2018 and 2020 (*6*,*13*). To identify children with ASD, site personnel requested and linked records from health sources and education sources. All sites participating in 2022 had access to health and education records (Table 1). *International Classification of Diseases* (ICD), *Ninth Revision* (ICD-9) or ICD-10 developmental disability diagnosis codes were requested from health sources, which included service providers conducting developmental evaluations and state administrative programs. California, Pennsylvania, Puerto Rico, Utah, and Wisconsin had access to state-funded disability services programs or Medicaid claims data. Special education eligibility data were requested from education sources. Thirteen sites had data agreements in place with education sources covering 100% of their study areas; three sites had agreements with education data sources covering <100% (Georgia [97.3%], Missouri [68.0%], and Texas [Laredo] [99.7%]). California, Maryland, New Jersey, Pennsylvania, Puerto Rico, Utah, and Wisconsin had access to IDEA Part C early intervention data. Certain data sources (e.g., Medicaid) are administrative in nature and did not have physical or electronic records such as developmental evaluations available for review. Indiana had access only to special education eligibility category data from its educational source and ICD codes from its medical source. Therefore, Indiana was not included in analyses or visualizations of elements of the ASD case definition because information about ASD diagnostic statements was not available.

**TABLE 1:** Prevalence of autism spectrum disorder per 1,000 children aged 8 years with descriptions of surveillance sites and data sources — Autism and Developmental Disabilities Monitoring Network, 16 sites, United States, 2022

Site	Surveillance area description	Types of data sources used*	% population coverage of education data sources^†^	% of cases with records available for abstraction at ≥1 source^§^	Total population	No. with ASD	ASD prevalence (95% CI)^¶^
Arizona	Part of one county in metropolitan Phoenix	Health and education	100	100	6,709**	210	31.3 (27.4–35.7)
Arkansas	21 counties in central Arkansas	Health and education	100	99.8	15,319	457	29.8 (27.3–32.6)
California	Part of one county in metropolitan San Diego	Health, education, early intervention, and state developmental disability services	100	100	15,212**	807	53.1 (49.6–56.7)
Georgia	Three counties in metropolitan Atlanta	Health and education	97.3	80.3	35,213	1,149	32.6 (30.8–34.5)
Indiana	One county in metropolitan Indianapolis	Health and education	100	—^††^	13,155	241	18.3 (16.2–20.8)
Maryland	Five counties in the Baltimore area	Health, education, and early intervention	100	99.8	21,206	558	26.3 (24.2–28.6)
Minnesota	Parts of three counties in the Twin Cities metropolitan area	Health and education	100	91.2	17,331**	616	35.5 (32.9–38.4)
Missouri	Three counties in metropolitan St. Louis	Health and education	68.0	100	19,968	640	32.1 (29.7–34.6)
New Jersey	Two counties in the New York metropolitan area	Health, education, and early intervention	100	97.9	18,334	623	34.0 (31.5–36.7)
Pennsylvania	One county in suburban Philadelphia	Health, education, early intervention, and Medicaid claims including state-funded long-term care programs	100	83.9	7,066	335	47.4 (42.7–52.6)
Puerto Rico	32 municipalities in north, east, south, and central regions of Puerto Rico	Health, education, early intervention, Medicaid claims, and autism registry	100	98.7	17,457	461	26.4 (24.1–28.9)
Tennessee	11 counties in middle Tennessee	Health and education	100	88.8	26,182	889	34.0 (31.8–36.2)
Texas (Austin^§§^)	Part of one county in south central Texas	Health and education	100	84.7	4,356**	85	19.5 (15.8–24.1)
Texas (Laredo)	One county in south Texas	Health and education	99.7	83.0	4,856	47	9.7 (7.3–12.8)
Utah	Three counties in northern Utah	Health, education, early intervention, and habilitative services	100	89.8	24,395	658	27.0 (25.0–29.1)
Wisconsin	Eight counties in southeastern Wisconsin	Health, education, early intervention, Medicaid claims, and state-funded long-term care program	100	77.8	28,098	1,078	38.4 (36.2–40.7)
**Total**				**90.9^¶¶^**	**274,857**	**8,854**	**32.2 (31.6–32.9)**

**Abbreviations:** ASD = autism spectrum disorder.

* Health sources include records from medical and service providers that evaluate children with developmental disabilities.

^†^ For public schools in the surveillance area.

^§^ The percentage of children with electronic or physical records available from ≥1 source for abstraction. Certain data sources (e.g., Medicaid) are administrative in nature and do not have physical or electronic records such as developmental evaluations available for review.

^¶^ 95% CIs were calculated using the Wilson score method.

** Denominator excludes tracts that were not included in the surveillance area using American Community Survey data.

^††^ Indiana did not have data from record abstraction available.

^§§^ Site name reflects the location of the surveillance team and not the surveillance area.

^¶¶^ The total number of cases with records available for abstraction at ≥1 source was 7,831 and total number of cases from sites with data from record abstraction available was 8,613.

For this report, children aged 8 years (born in 2014) or aged 4 years (born in 2018) met the surveillance ASD case definition if they lived in the surveillance area at any time in 2022 and they received 1) an ASD diagnostic statement in a comprehensive developmental evaluation, 2) autism special education eligibility, or 3) an ASD ICD-9 code in the 299 range or ICD-10 code of F84.0, F84.3, F84.5, F84.8, or F84.9. Children aged 4 years were classified as having suspected ASD if they did not meet the criteria for ASD but had an evaluator’s suspicion of ASD documented in a comprehensive developmental evaluation. Additional demographic information, comprehensive developmental evaluations, individualized education programs (IEPs), scores from intelligence quotient (IQ) assessments, and presence of ASD diagnostic tests and assessment tools (tests) were collected from records for children.

### Additional Data Sources and Variable Definitions

The numbers of children aged 4 and 8 years living in each surveillance area were obtained from the U.S. Census Bureau Vintage 2022 county-level single-year-of-age postcensal population estimates for 2022 (https://www.census.gov/programs-surveys/popest/technical-documentation/methodology.html). Surveillance areas at four sites (Arizona, California, Minnesota, and Texas [Austin]) were partial counties and were defined using census tracts. For these sites, postcensal population estimates were adjusted using American Community Survey (ACS) estimates of included census tracts (*14*). Full details are available (Supplementary Methods 1 and Supplementary Table 1, https://stacks.cdc.gov/view/cdc/177099#tabs-3). 

When information on race and ethnicity or sex was missing from records, birth certificate data were used if available. Co-occurring intellectual disability was defined as an IQ score of ≤70 or an examiner’s statement of intellectual disability on the child’s most recent IQ test. Evaluation by age 36 months was calculated using the earliest recorded developmental evaluation for each child. Earliest age at identification was defined as the child’s age in months at first recorded ASD diagnosis or special education eligibility (identification ages for children with only an ICD code were not available). Children were linked at the census tract level to socioeconomic indicators of neighborhood MHI from the 2022 ACS 5-year estimates (*14*) and CDC’s social vulnerability index (SVI) (*15*).

### Analytic Methods

ASD prevalence among children aged 8 years was calculated as the number of children who met the ASD surveillance case definition per 1,000 children of that age living in the surveillance area; ASD prevalence among children aged 4 years was calculated similarly. A Bayesian hierarchical modelling approach and random-effects restricted maximum likelihood method were also used to calculate overall prevalence and measures of uncertainty (95% Bayesian credible intervals and 95% CIs, respectively) among children aged 8 years to better account for the wide range in prevalence across sites; full details are available (Supplementary Methods 2, https://stacks.cdc.gov/view/cdc/177099#tabs-3). Prevalence was calculated by sex and by race and ethnicity for American Indian or Alaska Native (AI/AN), Asian or Pacific Islander (A/PI), non-Hispanic Black (Black), non-Hispanic White (White), multiracial (two or more races), and Hispanic or Latino (Hispanic) children. Children of Hispanic origin of any race were categorized as Hispanic; all other racial groups were categorized as non-Hispanic. Children lacking information on sex (n = 13 children aged 8 years) or race and ethnicity (n = 106 children aged 4 years and 115 children aged 8 years) were excluded from analyses stratified by those variables. The U.S. Census Bureau’s Population Estimates Program does not include race and Hispanic origin detail for Puerto Rico at the municipio (municipality) level. Of the overall Puerto Rico population, 99% is Hispanic (*16*); for this analysis, children aged 4 and 8 years in Puerto Rico were considered Hispanic. Denominators for prevalence were therefore not available for cases with non-Hispanic ethnicity (n = 4 children aged 4 years and n = 2 children aged 8 years) reported by Puerto Rico.

Census tracts for all sites combined were grouped into low, medium, and high tertiles for MHI that included roughly equal populations of children in each respective age group. For children aged 8 years, the low tertile included neighborhoods with MHI up to $62,470, medium tertile up to $97,768, and high tertile up to $250,001. SVI data were grouped into low, medium, and high tertiles based on national percentile. Prevalence of ASD calculated by MHI and SVI tertile used the appropriate ACS 5-year population denominator (population of group aged 5–9 years divided by five to estimate a single year of age) for census tracts included in each tertile.

Prevalence estimates with a relative standard error ≥30% were suppressed because of limited statistical precision. Prevalence ratios were used to compare prevalence by sex and by race and ethnicity; prevalence ratios involving at least one suppressed estimate were likewise suppressed.

Because Indiana did not have data available from record abstraction, Indiana is included in analyses of prevalence (by demographic characteristics, MHI, and SVI) but excluded from other analyses that required data from record abstraction (e.g., co-occurring intellectual disability or presence of evaluation or diagnosis or age at evaluation or diagnosis).

Cumulative incidence of ASD per 1,000 children was calculated separately for children aged 4 and 8 years in 2022 by dividing the total number of children with earliest ASD diagnosis or eligibility at each month of age by the population denominator for children aged 4 years or 8 years in 2022. Cumulative incidence of diagnosis or eligibility by age 48 months was compared between children born in 2018 (aged 4 years in 2022) and those born in 2014 (aged 8 years in 2022) using risk ratios.

To assess the effect of potential service disruption during the COVID-19 pandemic on patterns of evaluation and identification, numbers of evaluations and identifications were aggregated by calendar month for children aged 4 and 8 years in 2022. To compare the same age windows (age 0–4 years) by calendar month, the numbers of evaluations and incidence of identification per 1,000 children from 2014 (year 0) through 2018 (year 4) for children aged 8 years was subtracted from the same months during 2018 (year 0) through 2022 (year 4) for children aged 4 years.

The Wilson score method was used to calculate 95% CIs for prevalence, prevalence ratios, prevalence differences, cumulative incidence, and risk ratios. Prevalence and risk ratios were considered significant when 95% CIs did not include 1.0. Prevalence differences (comparing 2020 and 2022 for sites participating in both years) were considered significant when 95% CIs did not include 0. The male-to-female prevalence ratio was compared among sites using the Mantel-Haenszel test of homogeneity. Cochran-Armitage tests of trend were used to assess trends across MHI and SVI tertiles when data were available for all tertiles. Pearson chi-square tests were used to compare differences in distributions between groups for analyses of co-occurring intellectual disability and evaluation by age 36 months. Mantel-Haenszel, Cochran-Armitage tests of trend, and chi-square tests were considered significant when the p value was <0.05. R software (version 4.4.1; R Foundation) was used for data analysis and visualization.

## Results

### ASD Prevalence Among Children Aged 8 Years

The overall observed ASD prevalence was 32.2 per 1,000 (one in 31) children aged 8 years and ranged from 9.7 in Texas (Laredo) to 53.1 in California (Table 1). The overall ASD prevalence using the Bayesian hierarchical modelling approach was 32.2 (95% Bayesian credible interval = 25.4–39.3) per 1,000 children aged 8 years; with the random-effects restricted maximum likelihood method it was 30.9 (95% CI = 25.8–36.0) per 1,000 children aged 8 years (Supplementary Methods 2, https://stacks.cdc.gov/view/cdc/177099#tabs-3). Eleven sites also conducted ASD surveillance in 2020; estimates and differences in site boundaries or access to data sources are available (Supplementary Table 2, https://stacks.cdc.gov/view/cdc/177099#tabs-3). Among the 11 sites, prevalence was higher in 2022 than in 2020 at nine (from 14.0% to 36.7% higher) and overall (22.2% higher [absolute difference of 6.1 more children with ASD per 1,000 children aged 8 years]). Prevalence was similar between 2020 and 2022 at two sites (Arizona and Utah). Further limiting the comparison to the five sites where site boundaries and data sources were the same between years, the absolute increase in ASD prevalence from 2020 to 2022 was still 6.1 per 1,000 children aged 8 years (22.8% higher) (Supplementary Table 2, https://stacks.cdc.gov/view/cdc/177099#tabs-3).

The overall male-to-female prevalence ratio was 3.4, with overall ASD prevalence of 49.2 per 1,000 among boys and 14.3 per 1,000 among girls (Table 2). Evidence of heterogeneity of the male-to-female ratio was observed across sites (Table 2). ASD prevalence among children aged 8 years differed by racial and ethnic groups (calculated as ratios with White children, observed to have the lowest prevalence, as the reference group) (Table 2); prevalence ratios for additional comparisons of racial and ethnic groups are presented (Supplementary Table 3, https://stacks.cdc.gov/view/cdc/177099#tabs-3). Prevalence among White children (27.7) was lower than prevalence among multiracial (31.9), Hispanic (33.0), Black (36.6), AI/AN (37.5), or A/PI children (38.2). Utah was the only site in which another racial or ethnic group had lower ASD prevalence than White children (multiracial compared with White).

**TABLE 2:** Prevalence of autism spectrum disorder per 1,000 children aged 8 years, by sex* and race and ethnicity^†^ — Autism and Developmental Disabilities Monitoring Network, 16 sites, United States, 2022

Site	Prevalence by sex (95% CI)^§^		Prevalence ratio (95% CI)^§^	Prevalence by race (95% CI)^§^					Prevalence ratio (95% CI)^§^			
	Male	Female	Male to female^¶^	A/PI	Black	Hispanic	Multiracial	White	A/PI to White	Black to White	Hispanic to White	Multiracial to White
Arizona	48.3 (41.6–56.0)	13.7 (10.2–18.2)	3.5 (2.6–4.9)**	27.1 (15.2–47.9)	—^††^	32.2 (25.6–40.5)	—^††^	33.4 (27.6–40.5)	0.8 (0.4–1.5)	—^††^	1.0 (0.7–1.3)	—^††^
Arkansas	48.1 (43.5–53.0)	10.8 (8.7–13.4)	4.4 (3.5–5.6)**	51.2 (28.8–89.3)	29.9 (24.8–35.9)	28.9 (21.5–38.8)	24.7 (15.7–38.7)	29.5 (26.2–33.1)	1.7 (1.0–3.1)**	1.0 (0.8–1.3)	1.0 (0.7–1.4)	0.8 (0.5–1.3)
California	80.1 (74.4–86.3)	23.1 (19.9–26.8)	3.5 (2.9–4.1)**	56.8 (47.1–68.4)	65.4 (51.7–82.4)	54.1 (49.3–59.4)	67.2 (53.8–83.7)	41.4 (35.4–48.4)	1.4 (1.1–1.8)**	1.6 (1.2–2.1)**	1.3 (1.1–1.6)**	1.6 (1.2–2.1)**
Georgia	51.1 (47.9–54.4)	13.7 (12.1–15.5)	3.7 (3.2–4.3)**	35.6 (29.8–42.4)	39.1 (36.1–42.3)	27.7 (23.9–32.0)	26.1 (19.1–35.4)	24.0 (21.0–27.4)	1.5 (1.2–1.8)**	1.6 (1.4–1.9)**	1.2 (0.9–1.4)	1.1 (0.8–1.5)
Indiana	27.5 (23.8–31.7)	8.7 (6.7–11.3)	3.2 (2.3–4.2)**	37.7 (26.4–53.6)	15.5 (12.2–19.5)	19.4 (14.5–25.9)	—^††^	18.2 (14.8–22.4)	2.1 (1.4–3.1)**	0.9 (0.6–1.2)	1.1 (0.7–1.5)	—^††^
Maryland	41.3 (37.7–45.2)	10.7 (8.9–12.9)	3.9 (3.1–4.7)**	31.3 (24.5–40.0)	36.8 (32.0–42.3)	28.6 (22.4–36.4)	29.5 (21.3–40.8)	19.3 (16.9–22.1)	1.6 (1.2–2.1)**	1.9 (1.6–2.3)**	1.5 (1.1–2.0)**	1.5 (1.1–2.2)**
Minnesota	53.6 (49.1–58.5)	17.4 (14.9–20.4)	3.1 (2.6–3.7)**	32.6 (26.2–40.5)	41.1 (35.5–47.4)	40.9 (32.8–50.8)	46.0 (35.3–59.8)	29.6 (26.0–33.6)	1.1 (0.9–1.4)	1.4 (1.1–1.7)**	1.4 (1.1–1.8)**	1.6 (1.2–2.1)**
Missouri	47.8 (43.8–52.1)	15.7 (13.4–18.3)	3.1 (2.5–3.7)**	55.1 (41.4–72.9)	35.2 (30.6–40.4)	26.3 (18.1– 38.0)	24.9 (16.9–36.5)	29.1 (26.2–32.3)	1.9 (1.4–2.6)**	1.2 (1.0–1.4)**	0.9 (0.6–1.3)	0.9 (0.6–1.3)
New Jersey	52.9 (48.5–57.6)	14.3 (12.0–16.9)	3.7 (3.1–4.5)**	26.4 (18.9–36.9)	34.8 (30.2–40.1)	40.7 (36.1–45.8)	36.3 (23.1–56.6)	23.5 (19.6–28.2)	1.1 (0.8–1.6)	1.5 (1.2–1.9)**	1.7 (1.4–2.1)**	1.5 (0.9–2.5)
Pennsylvania	72.4 (64.4–81.3)	21.6 (17.3–27.0)	3.4 (2.6–4.3)**	44.9 (30.4–66.0)	57.5 (48.0–68.9)	48.1 (32.5–70.6)	52.9 (34.5–80.3)	41.0 (35.1–47.9)	1.1 (0.7–1.7)	1.4 (1.1–1.8)**	1.2 (0.8–1.8)	1.3 (0.8–2.0)
Puerto Rico	39.8 (35.9–44.1)	12.5 (10.4–15.1)	3.2 (2.6–3.9)**	—^§§^	—^§§^	26.3 (24.0–28.8)	—^§§^	—^§§^	—^§§^	—^§§^	—^§§^	—^§§^
Tennessee	51.6 (48.0–55.5)	15.3 (13.3–17.6)	3.4 (2.9–3.9)**	51.0 (38.2–67.8)	38.5 (33.3–44.6)	41.1 (35.1–48.1)	29.1 (21.5–39.3)	29.4 (26.9–32.1)	1.7 (1.3–2.3)**	1.3 (1.1–1.6)**	1.4 (1.2–1.7)**	1.0 (0.7–1.4)
Texas (Austin)	28.5 (22.4–36.2)	9.6 (6.2–14.8)	3.0 (1.8–4.9)**	—^††^	—^††^	21.8 (17.4–27.5)	—^††^	—^††^	—^††^	—^††^	—^††^	—^††^
Texas (Laredo)	15.9 (11.7–21.6)	3.0 (1.4–6.2)	5.3 (2.4–11.8)**	—^††^	—^††^	10.0 (7.5–13.3)	—^††^	—^††^	—^††^	—^††^	—^††^	—^††^
Utah	39.9 (36.6–43.5)	12.3 (10.5–14.5)	3.2 (2.7–3.9)**	27.4 (19.3–38.6)	45.7 (29.4–70.2)	28.5 (24.3–33.4)	12.4 (7.4–20.7)	25.4 (23.1–28.0)	1.1 (0.8–1.5)	1.8 (1.1–2.8)**	1.1 (0.9–1.4)	0.5 (0.3–0.8)**
Wisconsin	57.5 (53.9–61.5)	17.9 (15.8–20.3)	3.2 (2.8–3.7)**	39.0 (30.6–49.4)	39.7 (34.5–45.6)	57.7 (51.4–64.8)	35.3 (26.8–46.2)	30.9 (28.3–33.7)	1.3 (1.0–1.6)**	1.3 (1.1–1.5)**	1.9 (1.6–2.2)**	1.1 (0.9–1.5)
**Total**	**49.2 (48.1–50.3)**	**14.3 (13.7–15.0)**	3.4 (3.3–3.6)**	**38.2 (35.4–41.2)**	**36.6 (35.1–38.1)**	**33.0 (31.7–34.4)**	**31.9 (29.0–35.1)**	**27.7 (26.8–28.7)**	1.4 (1.3–1.5)**	1.3 (1.3–1.4)**	1.2 (1.1–1.3)**	1.2 (1.0–1.3)**

**Abbreviations:** AI/AN = American Indian or Alaska Native; A/PI = Asian or Pacific Islander.

* Excludes children with unknown sex (n = 13).

^†^ Excludes children of other or unknown race (n = 115). Persons of Hispanic or Latino origin of any race are categorized as Hispanic; all racial groups are non-Hispanic. Overall AI/AN autism spectrum disorder prevalence per 1,000 was 37.5 (26.7–52.4). The Georgia site was the only site that met the threshold for statistical precision for AI/AN prevalence; the site-specific prevalence per 1,000 was 204.5 (111.5–345.0). AI/AN-to-White ratios were significant for Georgia (8.5 [95% CI = 4.7–15.5]) and overall (1.4 [95% CI = 1.0–1.9]).

^§^ 95% CIs were calculated using the Wilson score method.

^¶^ Mantel-Haenszel test of homogeneity of prevalence ratios across sites p<0.01, indicating heterogeneity in prevalence ratios across sites.

** Significant prevalence ratio (95% CI excludes 1.0).

^††^ Suppressed because relative standard error was ≥30% of the estimate or ratio involves at least one suppressed estimate.

^§§^ The U.S. Census Bureau Population Estimates Program does not include race and Hispanic origin detail for Puerto Rico. This methodology assumes that all Puerto Rico residents are Hispanic. Denominators were therefore not available for n = 2 children with ASD aged 8 years with non-Hispanic ethnicity reported by Puerto Rico.

**FIGURE 1:**
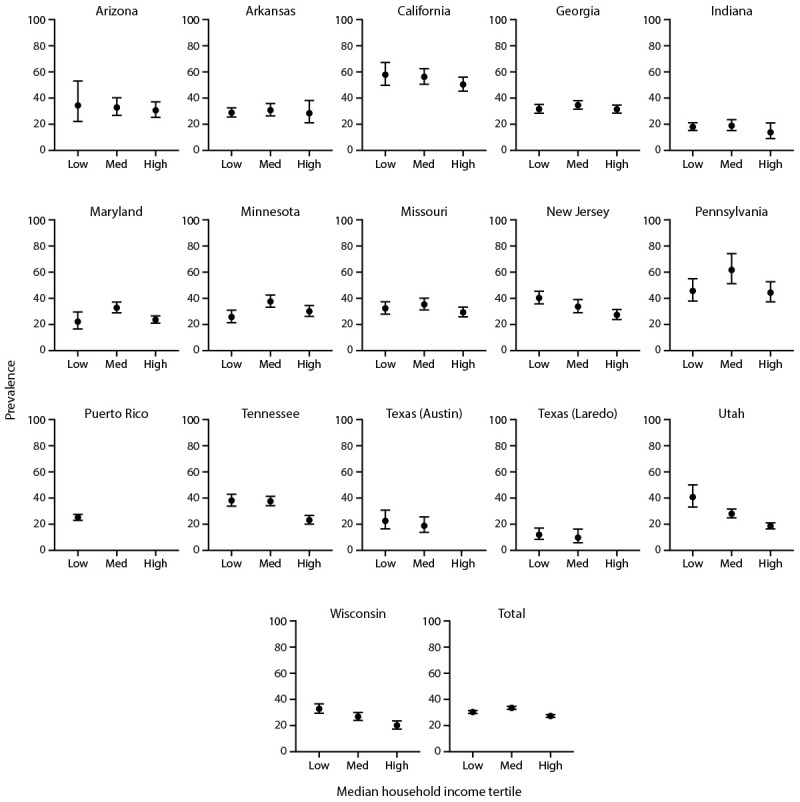
Prevalence* of autism spectrum disorder among children aged 8 years, by neighborhood median household income tertile and site^†^ — Autism and Developmental Disabilities Monitoring Network, 16 sites, United States, 2022^§^ **Abbreviation:** ASD = autism spectrum disorder; MHI = median household income. * Per 1,000 children aged 8 years. ^†^ Dots are point estimates and horizontal lines are 95% CIs. Neighborhood MHI tertiles = low ($2,499–$62,470), medium ($62,472–$97,768), high ($97,813–$250,001). Estimates for Puerto Rico medium and high MHI tertiles and Texas (Austin) and Texas (Laredo) high MHI tertiles were suppressed because relative standard error was ≥30% of the estimate. ^§^ Cochran-Armitage test of trend for association between MHI tertile and ASD prevalence, by site and overall: Arizona p = 0.6; Arkansas p = 0.8; California p = 0.1; Georgia p = 0.9; Indiana p = 0.5; Maryland p = 0.1; Minnesota p = 0.4; Missouri p = 0.3; New Jersey p<0.01; Pennsylvania p = 0.7; Puerto Rico p = 0.5; Tennessee p<0.01; Texas (Austin) p = 0.2; Texas (Laredo) p = 0.04; Utah p<0.01; Wisconsin p<0.01; Total p<0.01 (not monotonic). Ns and prevalence for each MHI tertile by site are available (Supplementary Table 4, https://stacks.cdc.gov/view/cdc/177099#tabs-3).

For 11 sites, ASD prevalence was not associated with neighborhood MHI, but lower neighborhood MHI was associated with higher ASD prevalence for five sites (New Jersey, Tennessee, Texas [Laredo], Utah, and Wisconsin) and overall (although the overall trend was not monotonic) (Figure 1) (Supplementary Table 4, https://stacks.cdc.gov/view/cdc/177099#tabs-3). Findings using SVI were generally similar: an association between SVI and ASD prevalence was not present at 11 sites and higher social vulnerability was observed with higher ASD prevalence at five sites (Maryland, New Jersey, Tennessee, Utah, and Wisconsin) and overall (although overall, Maryland and Tennessee trends were not monotonic) (Supplementary Figure 1, https://stacks.cdc.gov/view/cdc/177099#tabs-3).

### Presence of ASD Diagnostic Statements, Special Education Eligibility, and ICD Codes Among Children Aged 8 Years

**FIGURE 2:**
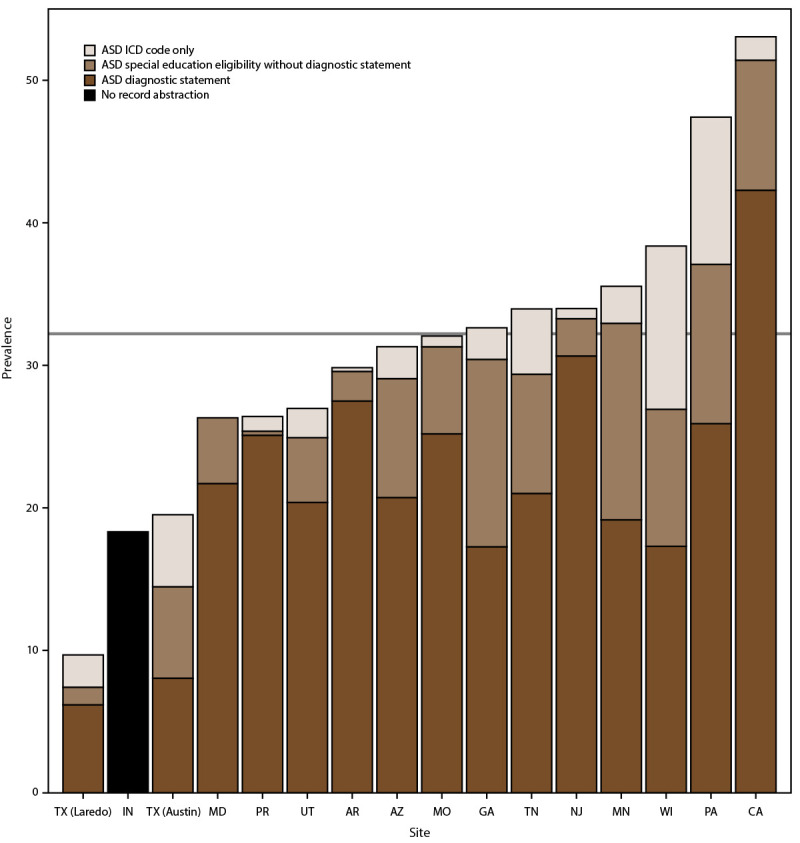
Prevalence* of autism spectrum disorder among children aged 8 years, by identification type and site^†^ — Autism and Developmental Disabilities Monitoring Network, 16 sites, United States, 2022^§^ **Abbreviations:** ASD = autism spectrum disorder; ICD = International Classification of Diseases. * Per 1,000 children aged 8 years. ^†^ Data from record abstraction were not available for Indiana. ^§^ Horizontal line is the overall Autism and Developmental Disabilities Monitoring Network prevalence of 32.2 per 1,000 children aged 8 years. Children with documented ASD statements could also have ASD eligibility in special education or ASD ICD codes. Underlying data are available (Supplementary Table 6, https://stacks.cdc.gov/view/cdc/177099#tabs-3).

**FIGURE 3:**
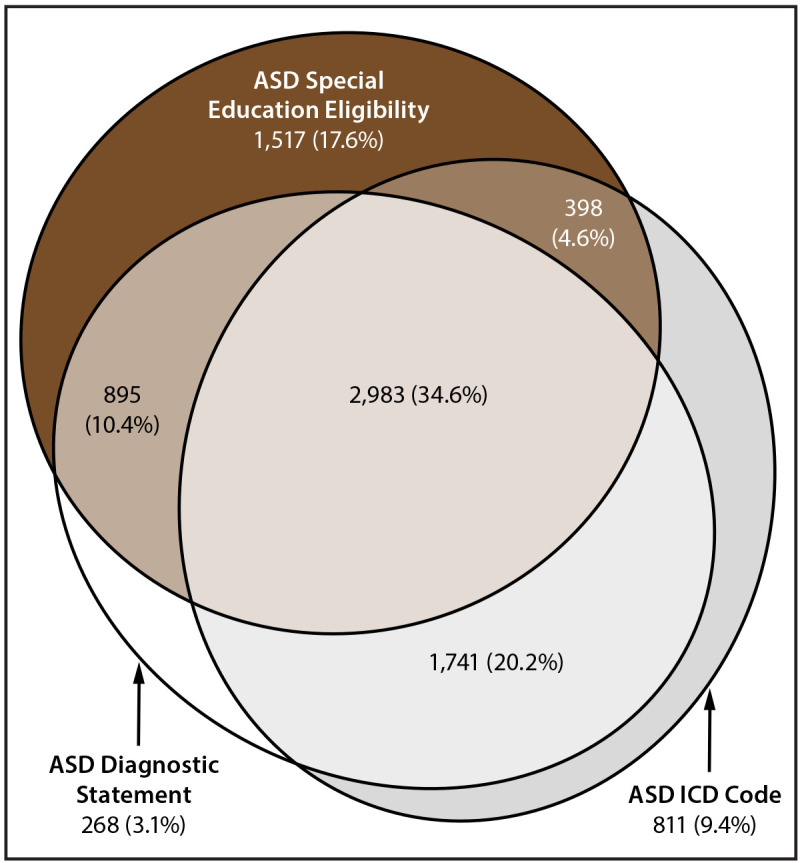
Euler diagram of different types of autism spectrum disorder identification among children aged 8 years with autism spectrum disorder* — Autism and Developmental Disabilities Monitoring Network, 15 sites, United States, 2022 **Abbreviations:** ASD = autism spectrum disorder; ICD = International Classification of Diseases. * N = 8,613 (the ADDM Network **has** 16 sites; Indiana is not included because the site did not have ASD diagnostic statement data from record abstraction available).

The percentage of children with diagnostic statements, special education eligibility, and ICD codes varied by site (Supplementary Tables 5 and 6, https://stacks.cdc.gov/view/cdc/177099#tabs-3). Among the 15 sites that completed record abstraction, the percentage of children with ASD who had a documented ASD diagnostic statement was 68.4% overall (range = 41.2% in Texas [Austin] to 95.0% in Puerto Rico) (Supplementary Table 5, https://stacks.cdc.gov/view/cdc/177099#tabs-3). ASD prevalence per 1,000 children aged 8 years based exclusively on documented ASD diagnostic statements was 22.5 overall (range = 6.2 in Texas [Laredo] to 42.3 in California) (Figure 2) (Supplementary Table 6, https://stacks.cdc.gov/view/cdc/177099#tabs-3). The overall percentage of children with ASD who had a documented autism special education eligibility was 67.3% (range = 38.3% in Texas [Laredo] to 90.2% in Puerto Rico) (Supplementary Table 5, https://stacks.cdc.gov/view/cdc/177099#tabs-3). The percentage of children with ASD who had a documented ICD code was 68.9% (range = 40.9% [Maryland] to 88.7% [Pennsylvania]). A majority (69.9%) of children with ASD had at least two of the three types of ASD identification documented in their records and 34.7% had all three types (Figure 3). Of 5,933 children with an ICD code, 86.3% also had a documented ASD diagnostic statement or autism special education eligibility; among all 8,613 children with ASD in the 15 sites that completed record abstraction, 9.4% met the case definition through having only an ICD code.

Categories of special education eligibility in children’s most recent IEPs varied by site; certain sites are in states that only use primary eligibility categories, whereas other sites also have information on secondary eligibility (Supplementary Table 7, https://stacks.cdc.gov/view/cdc/177099#tabs-3). Of children with ≥1 IEP available in their records (63.7% of children aged 8 years), the top five eligibility categories were autism (77.7% of children); speech or language impairment (24.7%); health, physical, or other disability (7.9%); developmental delay (6.9%); and intellectual disability (3.6%).

### Cognitive Ability Among Children with ASD Aged 8 Years

Data on cognitive ability were available for 5,292 (61.4%) children aged 8 years with ASD (range = 21.3% in Texas [Laredo] to 90.2% in Arkansas) (Table 3). Similar percentages of girls and boys with ASD had data on cognitive ability (61.0 and 61.7%, respectively). Black children were less likely to have data on cognitive ability (59.3%) than multiracial children (66.4%) and White children (63.1%). The median age of most recent cognitive test was 67 months overall and varied by site (range = 45 months in Texas [Austin] to 85 months in Puerto Rico) (Table 3). Among children aged 8 years with ASD who had data on cognitive ability, 39.6% were classified as having intellectual disability (IQ ≤70) at their most recent test or examination, 24.2% were classified in the borderline range (IQ = 71–85), and 36.1% were classified in the average or higher range (IQ >85) (Table 3). The percentage of children with cognitive data available who were classified as having intellectual disability varied widely among sites (range = 24.8% in Puerto Rico to 80.0% in Texas [Laredo]). Overall, a similar proportion of girls and boys with ASD had intellectual disability (40.4% versus 39.5%, respectively). By race and ethnicity, Black children had a higher proportion of co-occurring intellectual disability (52.8%) than all other groups except AI/AN (50.0%) children. A/PI (43.9%) and Hispanic (38.8%) children had higher proportions of co-occurring intellectual disability than multiracial (31.2%) and White children (32.7%).

**TABLE 3:** Availability and distribution of intelligence quotient scores among children aged 8 years with autism spectrum disorder, by site,* sex,^†^ and race and ethnicity^§^ — Autism and Developmental Disabilities Monitoring Network, 15 sites, United States, 2022

Site/Characteristic	Total no. with ASD	With IQ information	Median age of most recent IQ test (months)	Cognitive level		
		No. (%)		IQ ≤70 (%)	IQ 71–85 (%)	IQ >85^¶^ (%)
**Site**						
Arizona	210	163 (77.6)	82	51 (31.3)	48 (29.4)	64 (39.3)
Arkansas	457	412 (90.2)	76	179 (43.4)	87 (21.1)	146 (35.4)
California	807	658 (81.5)	64	187 (28.4)	179 (27.2)	292 (44.4)
Georgia	1,149	625 (54.4)	56	306 (49.0)	147 (23.5)	172 (27.5)
Maryland	558	426 (76.3)	77	186 (43.7)	114 (26.8)	126 (29.6)
Minnesota	616	436 (70.8)	72.5	163 (37.4)	91 (20.9)	182 (41.7)
Missouri	640	415 (64.8)	67	135 (32.5)	110 (26.5)	170 (41.0)
New Jersey	623	345 (55.4)	64	141 (40.9)	92 (26.7)	112 (32.5)
Pennsylvania	335	204 (60.9)	62	69 (33.8)	62 (30.4)	73 (35.8)
Puerto Rico	461	314 (68.1)	85	78 (24.8)	59 (18.8)	177 (56.4)
Tennessee	889	549 (61.8)	49	272 (49.5)	148 (27.0)	129 (23.5)
Texas (Austin)	85	54 (63.5)	45	38 (70.4)	—**	—
Texas (Laredo)	47	10 (21.3)	80.5	8 (80.0)	—	—
Utah	658	360 (54.7)	71.5	124 (34.4)	84 (23.3)	152 (42.2)
Wisconsin	1,078	321 (29.8)	58	161 (50.2)	52 (16.2)	108 (33.6)
**Total**	**8,613**	**5,292 (61.4)**	**67**	**2,098 (39.6)**	**1,282 (24.2)**	**1,912 (36.1)**
**Sex^††^**						
Female	1,868	1,140 (61.0)	66	460 (40.4)	282 (24.7)	398 (34.9)
Male	6,732	4,152 (61.7)	68	1,638 (39.5)	1,000 (24.1)	1,514 (36.5)
**Race and ethnicity^§§,^** ^¶¶^						
AI/AN	32	22 (68.8)	61	11 (50.0)	—	—
A/PI	627	394 (62.8)	68	173 (43.9)	89 (22.6)	132 (33.5)
Black	2,024	1,200 (59.3)	62	634 (52.8)	313 (26.1)	253 (21.1)
Hispanic	2,289	1,405 (61.4)	71	545 (38.8)	353 (25.1)	507 (36.1)
Multiracial	396	263 (66.4)	67	82 (31.2)	65 (24.7)	116 (44.1)
White	3,132	1,975 (63.1)	68	646 (32.7)	452 (22.9)	877 (44.4)

**Abbreviations:** AI/AN = American Indian or Alaska Native; A/PI = Asian or Pacific Islander; ASD = autism spectrum disorder; IQ = intelligence quotient.

* N = 8,613 (the ADDM Network includes 16 sites; Indiana is not included because the site did not have data from record abstraction available).

^†^ Excludes children with unknown sex (n = 13).

^§^ Excludes children of other or unknown race (n = 115). Persons of Hispanic origin might be of any race but are categorized as Hispanic; all racial groups are non-Hispanic.

^¶^ Includes 25 children stated to have an IQ score >70 but specific score was not given.

** Dashes indicate suppressed estimate because relative standard error was ≥30% of the estimate.

**^††^** Pearson chi-square test for proportion of males versus females with ASD and IQ information (p = 0.6); proportion of males versus females with ASD and IQ ≤70 (p = 0.6).

^§§^ Significant differences for Pearson chi-square tests for proportion of Black versus multiracial or White children with ASD and IQ information available (each comparison p = 0.01).

^¶¶^ Significant differences for Pearson chi-square tests for proportion of Black children with IQ ≤70 among children with ASD versus A/PI, Hispanic, multiracial, or White (each comparison p<0.001); A/PI children with IQ ≤70 among children with ASD versus multiracial or White (each comparison p<0.001); Hispanic children with IQ ≤70 among children with ASD versus multiracial (p = 0.02) or White children (p<0.001).

### Age at First Evaluation and ASD Diagnosis Among Children Aged 8 Years

Among 7,227 children aged 8 years with ASD and available evaluations, 50.3% were evaluated by age 36 months (range = 42.2% in Missouri to 63.6% in Pennsylvania) (Table 4). Among the 5,887 children aged 8 years with ASD who had an evaluation containing an ASD diagnostic statement, the median age at earliest known diagnosis was 47 months (range = 36 months in California to 69.5 months in Texas [Laredo]) (Table 4). Children with ASD and intellectual disability had a lower median age at diagnosis (43 months) than children without an intellectual disability (49 months).

**TABLE 4:** Number and percentage of children with developmental evaluations and median age at earliest documented autism spectrum disorder diagnosis among children aged 8 years, by site* — Autism and Developmental Disabilities Monitoring Network, 15 sites, United States, 2022

Site	Total no. with ASD	No. with evaluation (%)	No. with evaluation at ≤36 mos (%)	No. with diagnosis	Median age of earliest documented diagnosis among children with an ASD diagnostic statement (mos)		
					All	IQ ≤70^†^	IQ >70^†^
Arizona	210	191 (91.0)	104 (54.5)	139	45	38.5	47
Arkansas	457	457 (100.0)	228 (49.9)	421	53	48	60
California	807	801 (99.3)	448 (55.9)	643	36	36	35
Georgia	1,149	770 (67.0)	340 (44.2)	608	48	45	48.5
Maryland	558	546 (97.8)	337 (61.7)	460	48	38	52
Minnesota	616	514 (83.4)	250 (48.6)	332	52.5	43	63
Missouri	640	640 (100.0)	270 (42.2)	503	46	51	49.5
New Jersey	623	599 (96.1)	314 (52.4)	562	43	41	44
Pennsylvania	335	269 (80.3)	171 (63.6)	183	39	38	39
Puerto Rico	461	455 (98.7)	268 (58.9)	438	54	60	59.5
Tennessee	889	713 (80.2)	302 (42.4)	550	49.5	38	50
Texas (Austin)	85	67 (78.8)	41 (61.2)	35	42	38.5	46.5
Texas (Laredo)	47	32 (68.1)	—^§^	30	69.5	75	34
Utah	658	576 (87.5)	245 (42.5)	497	54	46	58.5
Wisconsin	1,078	597 (55.4)	315 (52.8)	486	43	38	50
**Total**	**8,613**	**7,227 (83.9)**	**3,638 (50.3)**	**5,887**	**47**	**43**	**49**

**Abbreviations:** ASD = autism spectrum disorder; IQ = intelligence quotient.

* N = 8,613 (the ADDM Network **has** 16 sites; Indiana is not included because the site did not have data from record abstraction available).

^†^ Includes only children with IQ information available.

^§^ Estimate suppressed because relative standard error was ≥30% of the estimate.

### Early ASD Identification Among Children Aged 4 and 8 Years

For 2022, prevalence per 1,000 children aged 4 years ranged from 12.9 (Indiana) to 60.6 (California) (Table 5) (Supplementary Figure 2, https://stacks.cdc.gov/view/cdc/177099#tabs-3). ASD prevalence per 1,000 children aged 4 years in the 16 sites combined was 29.3, which was 0.9 times the overall prevalence among children aged 8 years in 2022 (Table 5) (Supplementary Figure 3, https://stacks.cdc.gov/view/cdc/177099#tabs-3). Overall prevalence among children aged 4 years was lower than among children aged 8 years in seven sites (Arizona, Arkansas, Georgia, Indiana, Minnesota, Missouri, and Utah), similar to children aged 8 years in four sites (Maryland, Pennsylvania, Texas [Austin], and Wisconsin), and higher than among children aged 8 years in five sites (California, New Jersey, Puerto Rico, Tennessee, and Texas [Laredo]).

**TABLE 5:** Prevalence of autism spectrum disorder per 1,000 children aged 4 years compared with autism spectrum disorder prevalence among children aged 8 years, by surveillance site — Autism and Developmental Disabilities Monitoring Network, 16 sites, United States, 2022

Site*	Denominator for children aged 4 yrs	ASD cases among children aged 4 yrs	ASD prevalence among children aged 4 yrs (95% CI^†^)	ASD prevalence ratio comparing children aged 4 yrs with children aged 8 yrs (95% CI^†^)
Arizona	6,286	122	19.4 (16.3–23.1)	0.6 (0.5–0.8)^§^
Arkansas	14,644	360	24.6 (22.2–27.2)	0.8 (0.7–0.9)^§^
California	14,936	905	60.6 (56.9–64.5)	1.1 (1.0–1.3)^§^
Georgia	33,592	684	20.4 (18.9–21.9)	0.6 (0.6–0.7)^§^
Indiana	13,346	172	12.9 (11.1–14.9)	0.7 (0.6–0.9)^§^
Maryland	20,005	483	24.1 (22.1–26.4)	0.9 (0.8–1.0)
Minnesota	17,069	426	25.0 (22.7–27.4)	0.7 (0.6–0.8)^§^
Missouri	19,298	478	24.8 (22.7–27.1)	0.8 (0.7–0.9)^§^
New Jersey	18,260	665	36.4 (33.8–39.2)	1.1 (1.0–1.2)^§^
Pennsylvania	6,653	284	42.7 (38.1–47.8)	0.9 (0.8–1.1)
Puerto Rico	12,849	607	47.2 (43.7–51.0)	1.8 (1.6–2.0)^§^
Tennessee	26,363	958	36.3 (34.1–38.7)	1.1 (1.0–1.2)^§^
Texas (Austin)	4,405	74	16.8 (13.4–21.0)	0.9 (0.6–1.2)
Texas (Laredo)	4,357	62	14.2 (11.1–18.2)	1.5 (1.0–2.1)^§^
Utah	21,807	397	18.2 (16.5–20.1)	0.7 (0.6–0.8)^§^
Wisconsin	27,042	980	36.2 (34.1–38.5)	0.9 (0.9–1.0)
**Total**	**260,912**	**7,657**	**29.3 (28.7–30.0)**	**0.9 (0.9–0.9)^§^**

**Abbreviation:** ASD = autism spectrum disorder.

* Surveillance areas and data sources are the same for children aged 4 and aged 8 years.

^†^ 95% CIs were calculated using the Wilson score method.

^§^ Significant prevalence ratio (95% CI excludes 1.0).

Prevalence among children aged 4 years is also available by sex and race and ethnicity (Supplementary Table 8, https://stacks.cdc.gov/view/cdc/177099#tabs-3), as is information about components of the case definition (Supplementary Figure 4, https://stacks.cdc.gov/view/cdc/177099#tabs-3). Prevalence of suspected ASD was 3.1 per 1,000 children aged 4 years, which translated to one child suspected of having ASD for every 10 children identified with ASD among children aged 4 years (Supplementary Table 9 and Supplementary Figure 5, https://stacks.cdc.gov/view/cdc/177099#tabs-3).

**FIGURE 4:**
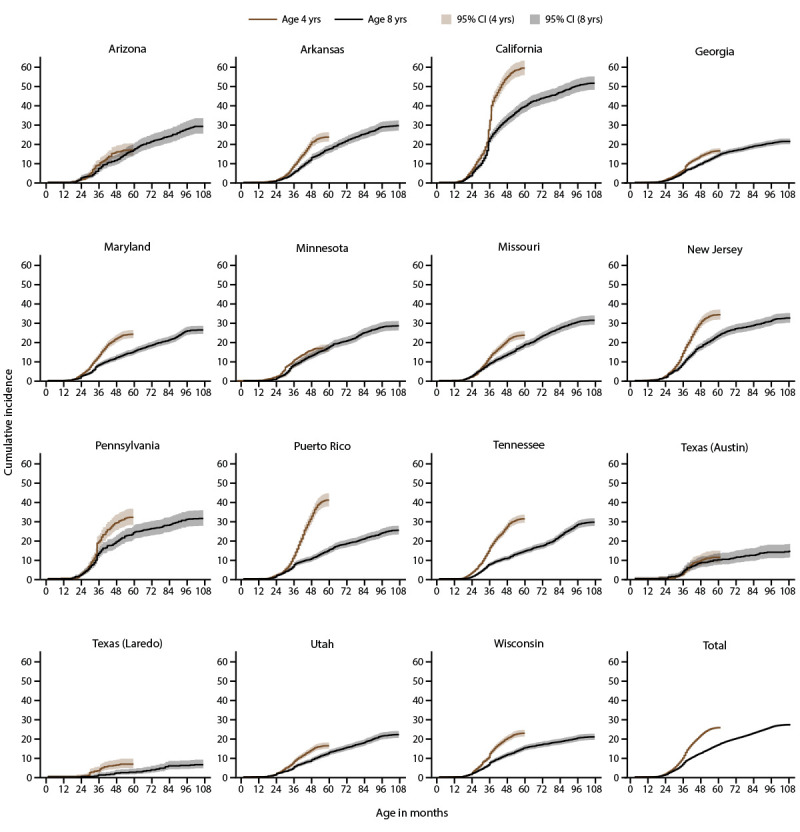
Cumulative incidence* of autism spectrum disorder diagnosis or autism special education eligibility among children aged 4 or 8 years, by month of age at identification and site^†^^,^^§^ — Autism and Developmental Disabilities Monitoring Network, 15 sites, United States, 2022^¶^ * Per 1,000 children aged 4 or 8 years. ^†^ The ADDM Network **has** 16 sites; Indiana is not included because the site did not have data from record abstraction available. ^§^ Not all children aged 4 years reach age 60 months and not all children aged 8 years reach age 108 months during the surveillance year. ^¶^ Data comparing cumulative incidence of autism spectrum disorder diagnosis or autism special education eligibility by age 48 months among children aged 4 or 8 years are available (Supplementary Table 10, https://stacks.cdc.gov/view/cdc/177099#tabs-3).

Children born in 2018 (aged 4 years in 2022) had 1.7 times the cumulative incidence of ASD diagnosis or eligibility by age 48 months as children born in 2014 (aged 8 years in 2022) (22.6 per 1,000 children compared with 13.1) (Figure 4) (Supplementary Table 10, https://stacks.cdc.gov/view/cdc/177099#tabs-3). This pattern was consistent at most (13 of 16) sites, ranging from 1.4 times as high in Arizona and Georgia to 3.1 times as high in Puerto Rico. Identification by age 48 months was similar between children born in 2014 and 2018 in Minnesota and Texas (Austin).

### Evaluation and Identification After COVID-19 Pandemic Onset

**FIGURE 5:**
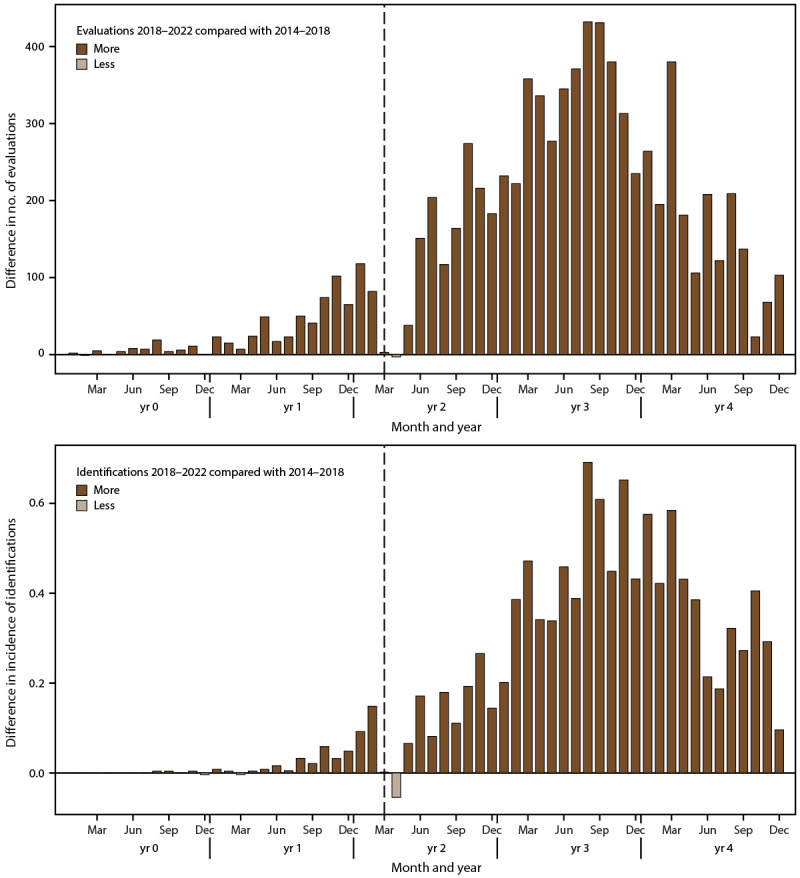
Difference in number of developmental evaluations and incidence* of autism spectrum disorder identification among children aged 4 years in 2022 during calendar years 2018–2022 and children aged 8 years in 2022 during calendar years 2014–2018, by month^†^**^,^**^§^ — Autism and Developmental Disabilities Monitoring Network, 15 sites, United States * Per 1,000 children aged 4 or 8 years. ^†^ The ADDM Network **has** 16 sites; Indiana is not included because the site did not have data from record abstraction available. ^§^ For children aged 4 years, year 0 to year 4 represents 2018–2022; for children aged 8 years, year 0 to year 4 represents 2014–2018. The dashed line shows the COVID-19 pandemic onset for children aged 4 years in 2022 compared with the analogous time window for children aged 8 years in 2022.

Children born in 2018 had more evaluations and ASD identifications than children born in 2014 when comparing the two groups of children for most months during the same age window (i.e., January 2018 through December 2022 for children aged 4 years in 2022 and January 2014 through December 2018 for children aged 8 years in 2022) (Figure 5). In March and April 2020, the first 2 months after the COVID-19 pandemic declaration, this pattern was disrupted, and the number of evaluations and rate of identification per 1,000 children was similar or lower for children born in 2018 compared with children born in 2014. The pattern of more evaluations and ASD identification among children born in 2018 resumed by June 2020 (Figure 5) and varied by site (Supplementary Figures 6 and 7, https://stacks.cdc.gov/view/cdc/177099#tabs-3).

### ASD Testing Among Children Aged 4 and 8 Years

**FIGURE 6:**
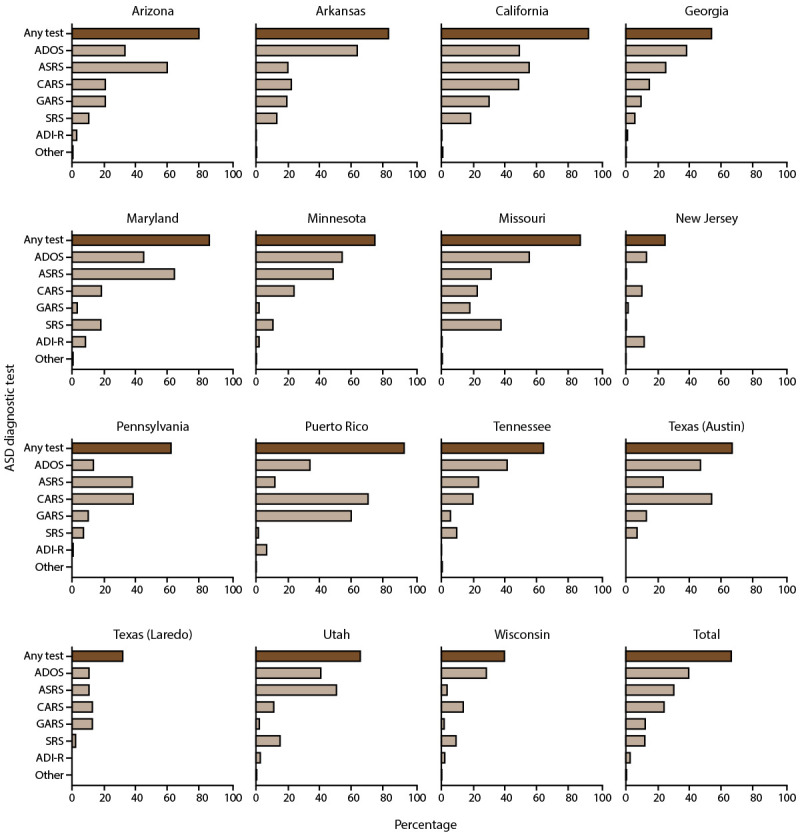
Percentage of children aged 8 years with autism spectrum disorder who have a recorded autism spectrum disorder diagnostic test, by site* — Autism and Developmental Disabilities Monitoring Network, 15 sites, United States, 2022 Abbreviations: **ADI-R = Autism Diagnostic Interview-Revised; ADOS = Autism Diagnostic Observation Schedule; ASD = autism spectrum disorder; ASRS = Autism Spectrum Rating Scales; CARS = Childhood Autism Rating Scale; GARS = Gilliam Autism Rating Scale; SRS = Social Responsiveness Scale; other test category includes Asperger Syndrome Diagnostic Scale, Gilliam Asperger’s Disorder Scale, and TELE-ASD-PEDS.** * N = 8,613 (the ADDM Network has 16 sites; Indiana is not included because the site did not have data from record abstraction available).

Autism testing practices varied among sites (Figure 6), ranging from 24.7% (New Jersey) to 93.5% (Puerto Rico) of children with ASD aged 8 years having any autism test documented in their records (Supplementary Table 11, https://stacks.cdc.gov/view/cdc/177099#tabs-3). Overall, 66.5% of children aged 8 years with ASD had any documented autism test. The most common autism tests were the Autism Diagnostic Observation Schedule (ADOS) (39.6% overall; range = 10.6%–63.9%), Autism Spectrum Rating Scales (ASRS) (30.2% overall; range = 0.3%–64.5%), Childhood Autism Rating Scale (CARS) (24.1% overall; range = 10.1%–70.7%), Gilliam Autism Rating Scale (GARS) (12.2% overall; range = 1.4%–60.1%), Social Responsiveness Scale (SRS) (12.0­­­­­­­% overall; range = 0.3%–37.7%), and Autism Diagnostic Interview-Revised (ADI-R) (2.7% overall; range = 0%–11.6%). Compared with children aged 8 years with ASD, a similar proportion of children aged 4 years with ASD had an ASD test documented in their records (69.1%), though the order of tests by frequency was different (Supplementary Figure 8, https://stacks.cdc.gov/view/cdc/177099#tabs-3). The most common autism test documented among children aged 4 years with ASD was the CARS (38.0%), followed by the ADOS (31.7%), ASRS (21.1%), TELE-ASD-PEDS (8.7%), SRS (8.1%), GARS (6.2%), and ADI-R (3.8%). ASD tests varied widely among children aged 4 years by site (Supplementary Figure 9, https://stacks.cdc.gov/view/cdc/177099#tabs-3).

## Discussion

In 2022, findings from the 16 diverse sites in the ADDM Network highlighted substantial differences in community practices for identifying ASD. ASD prevalence ranged widely, from 9.7 per 1,000 children aged 8 years in Texas (Laredo) to 53.1 in California. Variability was also observed in where children were identified (i.e., in a health or education setting), the frequency of cognitive and IQ testing, the proportion of children with IQ ≤70, and the use of ASD diagnostic tests. Variability in identification practices could affect receipt of services and suggests opportunities to implement successful identification strategies to ensure children in all communities receive the diagnostic and care services they need.

Research has not demonstrated that living in certain communities puts children at greater risk for developing ASD. Differences in the prevalence of children identified with ASD across communities might be due to differences in availability of services for early detection and evaluation and diagnostic practices. For example, California has had the highest prevalence among children aged 4 years and 8 years since joining the ADDM Network in 2018 (*6*,*10**,**11**,**13*) and has a local initiative (the Get SET Early model). As part of the initiative, hundreds of local pediatricians have been trained to screen and refer children for assessment as early as possible, which could result in higher identification of ASD, especially at early ages (*17*). In addition, California has regional centers throughout the state that provide evaluations and service coordination for persons with disabilities and their families (https://www.dds.ca.gov). Another reason for differences in prevalence could be whether children have insurance coverage or meet eligibility criteria for access to early intervention services. Pennsylvania, the site with second highest prevalence among children aged 8 years, has state Medicaid policy that includes children with physical, developmental, mental health, or intellectual disabilities regardless of parents’ income (*18*).

Puerto Rico was the site with second-highest prevalence among children aged 4 years, but prevalence among children aged 8 years in Puerto Rico was below the ADDM Network average. Higher ASD identification in the younger cohort could reflect dedicated joint efforts since 2017 by the Puerto Rico Title V Children with Special Health Care Needs Program and the Puerto Rico “Learn the Signs. Act Early.” Ambassador to decrease the age when children at risk for ASD receive their first diagnostic evaluation. These efforts have included developing and widely disseminating clinical protocols for early identification and diagnosis of ASD across Puerto Rico (*19*,*20*), increasing access to diagnostic evaluations at the Children with Special Health Care Needs Program Autism and Pediatric Centers for children aged ≤3 years, and providing all parents of newborns in Puerto Rico with a guide that includes information about developmental milestones and early ASD signs (*21*), among other activities.

Despite the variability in ASD prevalence across sites, a consistent pattern was observed of higher estimated ASD prevalence among A/PI, Black, Hispanic, and multiracial children than among White children across sites in 2022. This pattern was first observed among children aged 8 years in 2020 (*6*) and among children aged 4 years in 2018 (*10*) and contrasts with earlier ADDM findings that indicated the highest ASD prevalence was among White children compared with other groups (*7*).

Similarly, a previously reported pattern of higher ASD prevalence among children in higher SES neighborhoods from 2002 through 2010 (*7*) was last observed for one ADDM site in 2018 (*13*). The opposite pattern, higher ASD prevalence associated with low MHI, or no association of ASD prevalence with MHI, has been reported for other sites and overall from 2018 through 2022 (*6*,*13*). Use of SVI adds additional socioeconomic and community information (*15*) and similarly to MHI, higher ASD prevalence has not been associated with lower vulnerability at the site level and overall in 2020 (*22*) and 2022. Similar findings have been reported from the National Health Interview Survey, California Department of Developmental Services, and the England Spring School Census in recent years (*23*–*25*).

The reversal of these patterns in prevalence by race and ethnicity and SES is consistent with increased access to and provision of identification services among previously underserved groups. However, in a report examining ADDM Network data from 2020, higher MHI was still associated with higher ASD prevalence among A/PI, Black, and Hispanic children but not White children when stratified by both race and MHI, suggesting continued need for more equitable ASD identification (*22*). The low ASD prevalence observed in 2022 for both Texas sites, which included primarily Hispanic and lower MHI communities, could reflect this finding and suggest lack of access or barriers to accessing identification services.

Differences in health outcomes between racial and ethnic groups including higher rates of ASD and co-occurring intellectual disability could be related to differences in the frequency of social determinants of health (SDOH) characteristics (*26*). SDOH include individual and community-level factors such as low income, housing and food insecurity, and transportation barriers. Higher prevalence of intellectual disability (along with other neurologic disorders) might be related to higher rates of preterm birth, which is associated with brain injuries and neurodevelopmental impairment and also is linked to SDOH (*27*–*32*). In 2022, a total of 12.3% of births to Black mothers were preterm, compared with 8.7% of births to Hispanic and 7.6% of births to White mothers (*33*). Other causes of intellectual disability associated with SDOH include lead poisoning and traumatic brain injuries (*34*,*35*). SDOH could also contribute to disparities in access to early autism therapies, which have been found to increase cognitive and language scores (*36*).

ASD prevalence was consistently higher among boys than among girls across sites in 2022. Male-to-female prevalence ratios among children aged 8 years have narrowed in recent years from 4.2 in 2018 to 3.8 in 2020 to 3.4 in 2022 (*6*,*13*), but the decreasing prevalence ratio could be deceiving if interpreted as improvement in identification of girls with ASD. The difference in prevalence between boys and girls widened per 1,000 children from 27.7 in 2018 to 31.7 in 2020 to 34.9 in 2022.

Improvements over time in early identification have been apparent in the ADDM Network (*9*–*11*,*37*). In 2022, across the 13 sites with higher cumulative incidence of identification by age 48 months among children aged 4 years (born in 2018) compared with children aged 8 years (born in 2014), identification was 40%–300% higher in the younger group. At five sites, ASD prevalence was already higher among children aged 4 than among children aged 8 in 2022. These increases in early identification suggest services and supports for more persons with ASD across the lifespan could be needed in the future.

Evidence of a sustained effect of COVID-19 on early identification or evaluation for ASD in the 2022 ADDM Network cohorts was not apparent. Compared with the cohort born in 2014, the cohort born in 2018 had more evaluations and identifications before and after the pandemic from age 0–4 years; rates were similar for several months after the onset of the pandemic. The lack of sustained decreases could be related to the age of the children when they were affected by the pandemic. A telehealth assessment was found in records for 8.7% of the younger group, indicating telehealth could have helped children born in 2018 receive evaluations when they could not be performed in person because of COVID-19.

Prevalence of suspected ASD continued to be much lower than prevalence of identified ASD. This finding suggests that at the point children are receiving comprehensive developmental evaluations for concerns, few clinicians implement a “wait and see” approach. Because ADDM requests and reviews records from health sources administering comprehensive evaluations rather than from primary care providers, upstream disparities or delays in moving from suspicion to diagnosis at the primary care level could still exist. In October 2024, the Board of Directors of the American Academy of Pediatrics released a national payer advocacy letter calling for payers to allow general pediatricians to diagnose autism and to lift requirements for specific or repeated diagnostic evaluations (*38*). Eligibility for certain services can require an ASD diagnosis using specific instruments or processes (*39*). Removing such requirements could remove barriers for certain children to access ASD diagnoses or services while further increasing variability in how children are identified with ASD.

One reason suspected diagnoses could still be seen after comprehensive evaluation might be related to misperceptions about the age at which autism can be reliably diagnosed. For example, during review for the 2022 surveillance year one child with suspected ASD had the statement “autism suspected but cannot test until 4 years old” in their evaluation. However, autism can in certain cases be reliably identified as early as age 1 year (*40*). Developmental monitoring is important so that children with developmental disabilities including ASD can be evaluated, diagnosed, and supported as soon as possible. CDC’s “Learn the Signs. Act Early.” program provides a free milestone tracker app and developmental monitoring tools in multiple languages so parents, educators, and health care providers can monitor children’s development and address signs of delay early (https://www.cdc.gov/ncbddd/actearly).

## Limitations

The findings in this report are subject to at least five limitations. First, the populations within site-defined ADDM Network surveillance areas are not nationally representative and do not generate nationally representative ASD prevalence estimates. Reporting data for age 4 and age 8 years at the same time allows for comparisons between birth cohorts in the same surveillance period but surveillance areas and participating sites change over time, which can complicate comparisons. Second, overall estimates do not necessarily capture the full picture of variability across the many communities participating in the ADDM Network. Third, data quality is dependent on the availability and completeness of records at data sources, which vary by site and source. Certain data sources (e.g., Medicaid) do not have comprehensive evaluations available for abstraction. Demographic characteristics such as sex and race and ethnicity reflect what is documented in records rather than how families or persons might prefer to identify. Demographic categories could also vary between data sources and differ from U.S. Census Bureau race and ethnicity categories. Fourth, sources reviewed by the ADDM Network do not generally include private schools or primary care providers. Finally, the surveillance case definition of intellectual disability is not the same as a clinical diagnosis; IQ measurements in young children might lack stability and do not necessarily mean children have received a diagnosis of intellectual disability. Current diagnostic criteria for intellectual disability require documentation of adaptive behavior impairments, but adaptive assessments often are not present in children’s records.

## Future Directions

The ADDM Network will continue to monitor ASD prevalence among children aged 8 years, progress in early ASD identification among children aged 4 years, and the health status and transition needs among adolescents with ASD aged 16 years (*41*). Future reports could further apply hierarchical Bayesian modelling (or other) methods of aggregating data to reflect the uncertainty from combining widely varying estimates across communities. Linking additional and diverse data sources can expand geographic coverage of estimates and enrich knowledge of characteristics and needs of persons with ASD. Statewide data linkages could provide information about prevalence for more local communities for planning services (*42*). Information about post high-school outcomes for adolescents with autism could be obtained through linkages to explore factors that promote successful transition. These projects could help address data gaps for both emerging and prominent topics regarding ASD, such as supports and needs for adults, as well as better understanding of variability in testing and evaluation practices.

## Conclusion

Autism prevalence among children aged 8 years increased from 2020 to 2022. Prevalence in 2022 continued to vary widely across sites. Differences in prevalence over time and across sites can reflect differing practices in ASD evaluation and identification and availability and requirements that affect accessibility of services (e.g., meeting financial or diagnostic eligibility requirements). A/PI, Black, Hispanic, and multiracial children continued to have higher prevalence of ASD than White children, and children in low MHI or high vulnerability communities for five sites had higher prevalence of ASD than children in high MHI or low vulnerability communities. As evidence grows of increased access to identification among previously underserved groups, attention might shift to what factors, such as SDOH, could lead to higher rates of disability among certain populations. A higher rate of ASD identification by 48 months was found among children born in 2018 compared with children born in 2014. The cohort born in 2018 received more evaluations and ASD identifications than the cohort born in 2014 did during the same age window; disruption was visible at COVID-19 pandemic onset in early 2020 but the pattern of higher identification reappeared by the end of 2020. Continued increases in prevalence and improvements in early identification of ASD could indicate increasing need for services. Opportunities exist to learn from successful policies, systems, and practices in different communities and implement approaches for equitable identification or service eligibility to help families or persons receive the support they need as early as possible to improve outcomes for children with ASD.
